# Spatial roost networks and resource selection of female wild turkeys

**DOI:** 10.1098/rsos.231938

**Published:** 2024-05-29

**Authors:** Nicholas W. Bakner, Erin E. Ulrey, Patrick H. Wightman, Nick A. Gulotta, Bret A. Collier, Michael J. Chamberlain

**Affiliations:** ^1^ Warnell School of Forestry and Natural Resources, University of Georgia, Athens, GA 30602, USA; ^2^ School of Renewable Natural Resources, Louisiana State University Agricultural Center, Baton Rouge, LA 70803, USA

**Keywords:** avian, behaviour, resource selection, roost, spatial network, wild turkey

## Abstract

Wildlife demography is influenced by behavioural decisions, with sleep being a crucial avian behaviour. Avian species use roost sites to minimize thermoregulation costs, predation risk and enhance foraging efficiency. Sleep locations are often reused, forming networks within the home range. Our study, focusing on female eastern wild turkeys (*Meleagris gallopavo silvestris*) during the reproductive season, used social network analysis to quantify both roost site selection and network structure. We identified roost networks which were composed of a small percentage of hub roost sites connecting satellite roosts. Hub roosts were characterized by greater values of betweenness (*β* = 0.62, s.e. = 0.02), closeness (*β* = 0.59, s.e. = 0.03) and eigenvalue centrality (*β* = 1.15, s.e. = 0.05), indicating their importance as connectors and proximity to the network’s functional centre. The probability of a roost being a hub increased significantly with greater eigenvalue centrality. Female wild turkeys consistently chose roost sites at lower elevations and with greater topographical ruggedness. Hub roost probability was higher near secondary roads and further from water. Our research highlights well-organized roost site networks around hub roosts, emphasizing the importance of further investigations into how these networks influence conspecific interactions, reproduction and resource utilization in wild turkeys.

## 1. Introduction

Sleep behaviour is a prominent and vulnerable behavioural state that all wildlife species engage in [1–4]. Despite sleep behaviour being the most prominent behavioural state, little attention has been dedicated towards understanding aspects of sleep behaviours, such as where an individual chooses to sleep [3,5]. Behavioural decisions made while choosing a location to sleep are probably influenced by a host of abiotic and biotic factors as species are most vulnerable during sleep [3,6]. The dangers of sleep owing to increased vulnerability to predation are apparent; however, certain strategies can be considered safer than others and are more conducive to diurnal activities [6–8]. Thus, where an animal chooses to sleep plays a fundamental role in a species’ ecological processes [3].

Animals engage in spatial behaviours, moving among preferred habitat types or foraging sites, which develop into patterns like the route taken across the landscape with consequences in both spatial and social domains [9]. For diurnal species, sleeping sites can have critical consequences for individual fitness, as individuals must select sites that provide access to resources while providing protection from predators or natural elements [7,10]. Contemporary literature has noted that behavioural decisions such as sleep site switching [11,12] or reuse of sites may reduce predation risks and presumably influence individual fitness [13,14]. Understanding sleep behavioural decisions is important to fully comprehend their impact on ecological dynamics, including resource acquisition [15], mating [16] and mechanisms reducing predation risk [17]. Moreover, integrating social network analysis (SNA), which elucidates the spatial structure and implications for behaviour [18,19], allows for a more comprehensive evaluation of the consequences of spatial behaviours on social dynamics and network connectivity [20–22].

For avian species, roost sites are important as they reduce the cost of thermoregulation [23,24], decrease predation risk [25,26] and increase foraging efficiency [27–29]. Drivers of roost site selection are fundamental as an individual must position itself on the landscape in ways that are beneficial for resource acquisition, survival and reproduction [8]. Roost sites are ideally concentrated in core use areas, which may provide individuals with increased access to resources and reduced competition from conspecifics [30]. Hence, identifying the distribution of roosts within home ranges and how they are positioned relative to other roosts can allow an understanding of important ecological processes [31].

Roosting is an important aspect of wild turkey (*Meleagris gallopavo* spp.) ecology that can influence demography and spatial distribution [32]. The primary benefits of roosting are reduced predation risk and protection from adverse weather conditions [33,34]. Previous research on wild turkey roosting ecology has focused on roost tree descriptions and associated habitat conditions [35–37], and aspects of roost site selection at microhabitat or landscape scales [38–42]. Furthermore, site fidelity for male eastern wild turkeys (*M. gallopavo silvestris*; hereafter, wild turkeys) is low, suggesting that trees which provide suitable roost sites are abundant [43]. However, microhabitat and landscape characteristics at roosts vary across temporal and spatial scales, suggesting roost site selection may underlie access to receptive mates, foraging habitats and preferred vegetation cover used during nesting or brooding [32,38,43–47].

Reproduction drives wild turkey population trajectories, and female behavioural decisions have been shown to have a considerable influence on fitness outcomes [48–51]. During reproductive activities, female wild turkeys face strict spatial constraints, including access to males for breeding opportunities [52,53] and the need to stay near their nesting locations [48,50]. During the reproductive period, females transition from pre-laying areas to unfamiliar locations to initiate egg-laying [54]. Prospecting of unfamiliar areas is used to identify profitable locations during the egg-laying process to ensure there are adequate resources for incubation [55,56]. Thus, spatially quantifying roost selection and roost relationships for females throughout the reproductive period could offer insight into how they allocate themselves during sleep periods within these critical phenological periods.

Here, we used an SNA to evaluate roost site selection and structure of roost site networks for female eastern wild turkeys during the reproductive season. Specifically, we estimated site fidelity and identified hub and satellite roosts during different reproductive phases (i.e. pre-laying, laying, finished nesting and non-nesting), and we identified relevant landscape features selected at roost sites. We hypothesized that females would select roost sites to optimize their access to resources. Thus, we predicted females would have centralized hub roost sites (higher site fidelity) that exhibit higher degrees of fidelity in closer proximity to areas offering resources important during the reproductive season.

## 2. Methods

We used rocket nets to capture wild turkeys from January to March of 2014–2021 (for details on study sites refer to the electronic supplementary material, S1). We aged captured individuals based on the presence of barring on the ninth and tenth primary feathers and sexed them by the coloration of the breast feathers [57]. We banded each bird with an aluminium rivet leg band (National Band and Tag Company, Newport, Kentucky; female size = 8, male size = 9) and radio-tagged each individual with a backpack-style GPS-VHF transmitter [58] produced by Biotrack Ltd (Wareham, Dorset, UK). We programmed transmitters to record one GPS location nightly (23.58) and hourly GPS locations from 5.00 to 20.00 (Standard Time and according to the appropriate time zones) for the duration of the study [59]. Each transmitter had a mortality switch programmed to activate after more than 23 h of no movement. We released wild turkeys immediately at the capture location after processing. All wild turkey capture, handling and marking procedures were approved by the Institutional Animal Care and Use Committee at the University of Georgia (protocol nos. A2019 01-025-R2 and A2020 06-018-R1) and the Louisiana State University Agricultural Center (protocol nos. A2014-013, A2015-07 and A2018-13).

We performed data processing and analysis in program R (v. 4.1.0) [60]. We processed and cleaned the raw GPS data by removing locations that had dilution of precision values of greater than 7 [61]. To determine the dates of nest initiation (i.e. initiation of laying) and the onset of incubation, we mapped our spatial–temporal data using ArcGIS 10.8 (Environment Systems Research Institute, Redlands, CA, USA). We identified the onset of incubation as the first time an individual remained on the nest overnight [48,50], and then evaluated hourly locations for the previous 20 days to determine when a female initially visited the nest site (defined as location being <20 m from the known nest site [54,56,62]). We considered the date of the first visit as the date of nest initiation and used it as the beginning of the laying period, as wild turkeys rarely visit nest sites before laying the first egg [54,63]. We removed incubation locations from analyses because female wild turkeys remained on the ground at the nest location during this period. From the information described above, we were able to categorize reproductive phases for each female throughout the reproductive season, which we later used as a covariate in models to describe the temporal aspect of female roost selection ([64]; table 1).

**Table 1.** Descriptions of reproductive phases relative to roost site selection for female eastern wild turkeys (*M. gallopavo silvestris*).

reproductive phase	description
pre-laying	movements from 1 March until laying
laying	when a female initially visited the nest site and until incubation
finished nesting	when reproductive activity ceased
non-nester	females that did not make a nesting attempt

Wild turkeys may use specific roosting areas repeatedly but select different trees from one night to the next [32]. Therefore, we defined a roost site by running a sensitivity analysis of the distance between consecutive roost sites to provide an appropriate cluster radius (250 m) and considered any roost locations that fell within the cluster radius as a single roost site. We used the R package dbscan [65] to conduct the cluster analysis. We then estimated roost site fidelity (RF) as 1 minus the number of unique roost sites used divided by the number of nights within the period (RF = 1 − (unique roosts/total nights)) [43]. An RF value of 1 would indicate that an individual roosted in the same location every night, whereas values approaching 0 indicated that individuals roosted in a unique location every night. We calculated inter-roost distance between consecutive roosts for each female to identify phenological transitions in roosting behaviours across all birds [43,66]. For each roost site, we quantified elevation, slope, aspect and topographical ruggedness (degree of irregularity or roughness in the surface of a geographical area) using digital elevation models from the United States Geological Survey National Elevation Dataset (https://www.usgs.gov/the-national-map-data-delivery/gis-data-download, accessed 10 April 2023; [46,47,63]). We obtained road data for wildlife management areas (WMAs) from the Georgia Department of Natural Resources, Louisiana Department of Wildlife and Fisheries, South Carolina Department of Natural Resources and the United States Department of Agriculture: Forest Service and used United States Geological Survey Topologically Integrated Geographic Encoding and Referencing (TIGER)/Line data for roads outside the WMAs. We categorized roads as primary if they were paved/gravelled and vehicle access was not limited, whereas secondary roads were unpaved gravel and/or logging roads where vehicle use was reduced [67]. We obtained year-specific, 30 m resolution spatial data on landcover from the Cropland Data Layer (Cropscape) provided by the National Agricultural Statistics Service. We recoded and combined landcover in program R to create two unique landcover types (open treeless areas, water). We decided to exclude forested habitats in our analyses because wild turkeys will ultimately be in these areas because they roost in trees. Hence, we chose to use open treeless areas and water because these are resources that wild turkeys use for foraging opportunities [43,68]. We then calculated the nearest distance to primary and secondary roads and landcover types using the Euclidean distance tool in ArcGIS 10.8 (Environmental System Research Institute, Inc., Redlands, CA, USA) keeping all raster layers at a 30 m resolution.

### 2.1. Network analysis

Networks are composed of nodes connected by edges, which are used to understand network function and structure [19,69]. In our spatial network, we defined nodes as roost sites and assigned corresponding edges based on visits to different roost locations (roost-to-roost movement). Specifically, we used our RF values to define roost sites as it is more informative to use rates rather than raw counts [70]. We calculated a suite of metrics describing the nodes and edges for each individual female’s network (table 2). The distribution of the node degree can be used to determine the structure of a network, with the node degree being the number of edges one node has to other nodes [72]. In the context of roosting behaviour, degree is how connected a specific roost site is to the other roost sites within the network. Therefore, we calculated the degree distribution by summing the nodes that had edges and dividing by the total number of nodes.

**Table 2.** Descriptions of parameters used to describe spatial networks and the biological context of what information parameters provided for roost sites used by female eastern wild turkeys (*M. gallopavo silvestris*) [71].

parameters	description	biological context	biological relevance
betweenness	a count of the number of shortest paths that flow through the node	the number of times a roost forms a connection between other roost sites based on the shortest distance (Euclidian distance) from the roost site	this captures if an individual has a tendency to switch roost sites
closeness	the mean shortest path between a node and all other nodes	the shortest distance (Euclidian distance) between a roost and all other roosts within the network	identifies the spatial network positioning that can influence social dynamics, communication pathways, resource utilization and overall community resilience
clustering coefficient	a measure of cliquishness of the network, which is the fraction of a node’s immediate neighbours that are themselves neighbours	a measure of how close a roost is to other neighbouring roosts. This describes if there are communities of roosts that are not connected to the entire network	indicates the extent to which roosts are a community that can provide insight into social behaviour, resource utilization and overall fitness
degree	the number of edges that are connected to a node	the number of edges (connections) to a roost	this measure captures the association between other roost sites
edge	the interaction between two nodes	a female wild turkey moving between two roosts	the connectivity between roost sites
eigenvalue centrality	relative score assigned to nodes to assess their importance based on the connections to other nodes	assessment of the importance of a roost site in the network based on connections to other roost sites	this metric captures the potential importance of a roost site (hub) for propagation of information
node	an individual entity	the roost site	the roost site is used to understand the characteristics and attributes of a spatial network

We then characterized our nodes with few edges as satellite roost sites (lower degree) and those that have many edges as hub roost sites (high degree). We calculated betweenness, closeness, eigenvalue centrality and clustering coefficients for each unique roost cluster using the R package igraph ([73]; table 2). Betweenness, as defined by [74], suggests that a node (in this case, roost sites) plays a crucial role in connecting two other nodes within a network. In the context of roost sites, this implies that specific roosting locations have a significant influence on enhancing overall connectivity among different roosting sites. Closeness is a metric used to describe centrality which evaluates the shortest path length between a roost site and all other roost sites within the network [75]. Eigenvalue centrality describes the influence of a roost site by assessing the relative score of connected roosts with high-scoring roosts providing more importance than connections to low-scoring roosts [76]. Eigenvalue centrality is bounded from 0 and 1. The clustering coefficient evaluates cliques within a network, which means it identifies if there are localized communities (group) of roosts within the network. Using the 90th percentile values from the degree calculation, we determined whether roost sites were considered hubs or satellites [18,69]. Using the network metrics as covariates, we determined whether there were differences between hub and satellite roost sites. We tested for collinearity between each of our covariates and excluded covariates using Pearson’s correlation with an *r* > 0.60 [77]. We used a generalized linear model with a binomial response distribution and logit link to evaluate spatial network data for female wild turkeys. Our response was binary (0 = satellite, 1 = hub) to model network parameters at hub and satellite roosts. We used the R package glmmTMB to conduct our analysis [78].

### 2.2. Resource selection model

From the beginning of pre-laying until post-reproduction, we calculated 95% home ranges by fitting dynamic Brownian bridge movement models to the time-specific location data [59] using R package move [79]. We used an error estimate of 20 m, a moving window size of seven locations and a margin setting of three locations [59,80].

We used resource selection functions to examine relationships between distance to landcover types, distance to secondary roads and terrain features to wild turkey roost sites within individual home ranges (third-order selection) following a design III approach suggested by Manly *et al*. [81]. We tested for collinearity between each of our covariates and excluded covariates using Pearson’s correlation with an *r* > 0.60 [77]. After testing for collinearity, we removed the slope and primary roads from our models. We chose to retain topographical roughness, which is the standard deviation of the slope, instead of slope to characterize the relief of the terrain. We chose to keep secondary roads as they provide connectivity between areas selected by wild turkeys on our study sites as well as providing foraging opportunities [68]. We did not include interactions of aspect and reproductive phase as models failed to converge owing to quasi-complete separation. We compared each used point (roost site) to 50 available points sampled within each range [82]. We created four models which included a global model (i.e. including all covariates), a resource model, a feature model and a null model (table 3). Each model included an interaction of the reproductive phase for each female [64]. We used a generalized linear mixed model with a binomial response distribution (logistic regression) and logit link to the used-available data [81,83]. We used the glmmTMB R package [78] with a binary (0 = available, 1 = used) response variable to model resource selection [84]. To account for variability among individuals within our models, we included a random effect for each unique individual [84]. To improve performance and ease of interpretation, we rescaled all fixed effects by subtracting their mean and dividing by two standard deviations prior to modelling [85]. Similar to the methodology above, to determine if resource selection differed between hub and satellite roosts, we used a generalized linear mixed model with a binomial response distribution and logit link to evaluate spatial network data for female wild turkeys. Our response was binary (0 = satellite, 1 = hub) to model resources at hub and satellite roosts.

**Table 3.** Model structure for global, resource, feature and null models for female eastern wild turkey (*M. gallopavo silvestris*) roost sites during 2014–2021 across the southeastern United States. (Each covariate within the model had an interaction with reproductive phase (pre-laying, laying, finished nesting and non-nester). The resource covariates were calculated as a distance (m) to metric.)

model	parameter
global	secondary roads + open treeless area + water + elevation + ruggedness
resource	secondary roads + open treeless area + water
feature	elevation + ruggedness
null	(.)

We used second-order Akaike’s information criteria (AIC*c*) to assess the amount of support for the different candidate models [86,87]. We calculated ΔAIC*c* values between the AIC*c* value for candidate model *i* and the lowest-ranked AIC value. We also calculated Akaike’s weights (*w_i_*) for each model. We then calculated parameter estimates and their standard errors for all covariates in models within two ΔAIC*c* units of the lowest-ranked AIC value.

## 3. Results

We monitored 663 (560 adults, 102 juveniles, one unknown) female wild turkeys during 2014‒2021. We monitored 689 nesting attempts (initial attempts = 491, renesting attempts = 198) made by 499 females. We identified 66 364 roost locations after removing locations that occurred during incubation and brooding (table 4).

**Table 4.** Total and unique number of roost sites, mean site fidelity with associated standard deviations (s.d.) and mean inter-roost distance in meters with associated s.d. by reproductive phase for female eastern wild turkeys (*M. gallopavo silvestris*) across the southeastern United States during 2014–2021.

reproductive phase	total roost sites	unique roost sites	mean site fidelity (s.d.)	mean inter-roost distance (s.d.)
pre-laying	26 455	12 752	0.48 (0.19)	478.07 (505.95)
laying	7430	4242	0.41 (0.20)	554.46 (515.04)
finished nesting	21 114	9456	0.50 (0.21)	501.35 (478.68)
non-nester	11 365	5383	0.54 (0.19)	547.16 (545.86)

**Figure 1.**
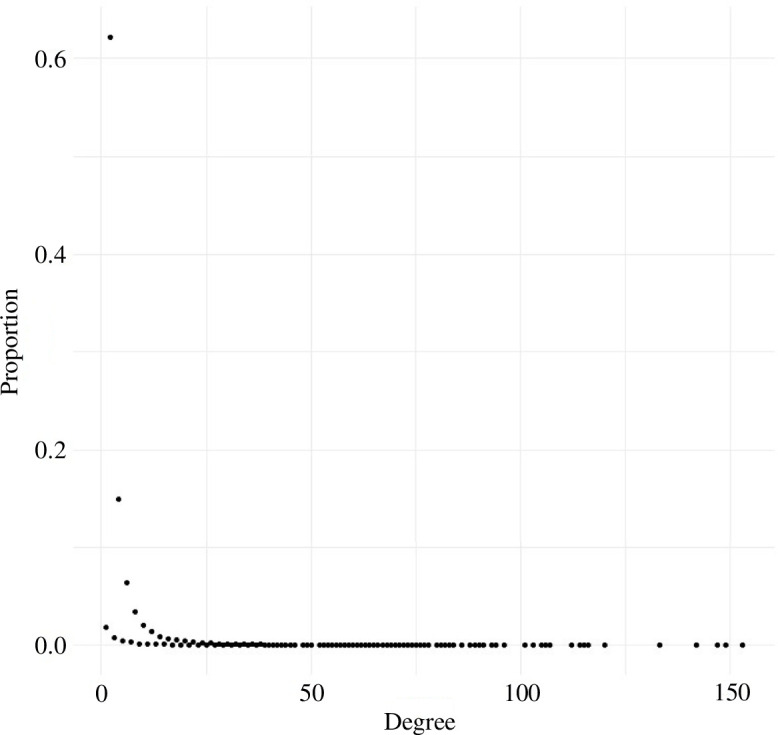
Degree distribution characterizing a spatial network for roost sites of female eastern wild turkeys (*M. gallopavo silvestris*) across the southeastern United States during 2014–2021.

**Figure 2.**
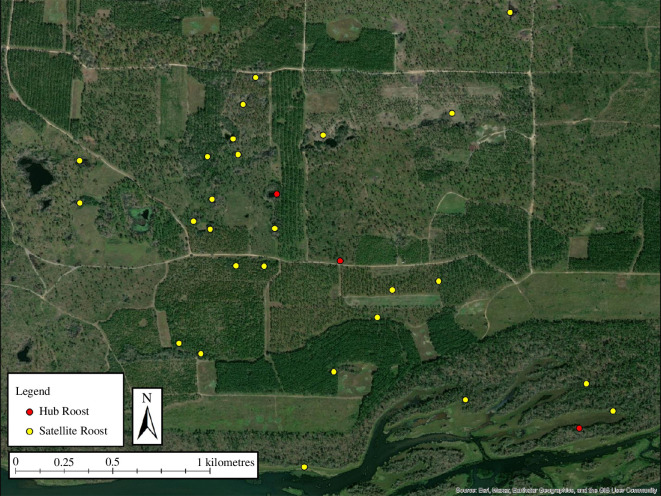
An example of a spatial network of roost sites for female eastern wild turkeys (*M. gallopavo silvestris*) across the southeastern United States during 2014–2021.

**Figure 3.**
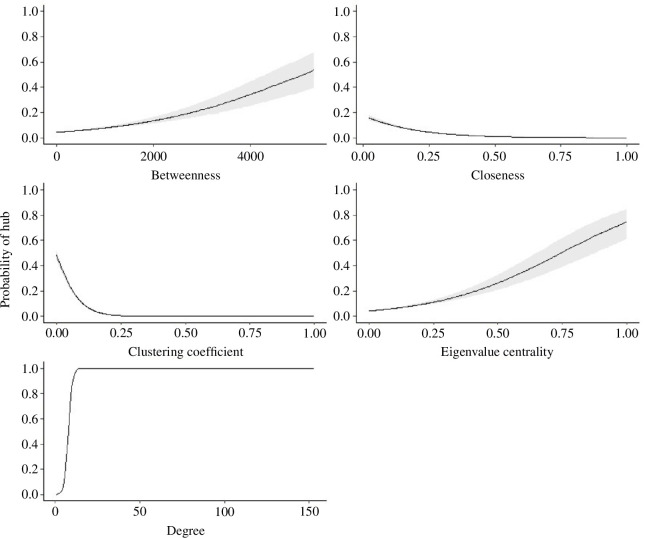
Predicted probability of hub roosts relative to betweenness, clustering coefficient, degree and eigenvalue centrality (solid line) with 95% confidence intervals (dotted line) from the best approximating model for female eastern wild turkeysy (*M. gallopavo silvestris*) across the southeastern United States during 2014–2021.

Our degree rate described individual female networks as having few (13.6% total roost sites) hub roost sites within their network (figure 1; table 5). Hub roosts had greater values of betweenness (*β* = 0.62, s.e. = 0.02), closeness (*β* = 0.59, s.e. = 0.03) and eigenvalue centrality (*β* = 1.15, s.e. = 0.05) but lower values of cluster coefficient (*β* = −0.36, s.e. = 0.02) than satellite sites (figure 2). Mean betweenness for hub roosts was 416 (s.d. = 334; table 5), suggesting that hub roosts served as connections or bridges within the network. Hub roosts were associated with higher closeness values suggesting that hub roosts were positioned near the functional centre of the network (figure 3). The probability of the roost site being a hub roost increased by 12.25% for every 0.1 increase in eigenvalue centrality, suggesting hub roosts played an important role in structuring the network (figure 2). Our clustering coefficient was lower for hub roosts suggesting one network rather than multiple communities.

**Table 5.** Descriptive statistics including median, mean, standard deviation and range for parameters included in a spatial network analysis of roost selection by female eastern wild turkeys (*M. gallopavo silvestris*) across the southeastern United States during 2014–2021.

parameter	median	mean	s.d.	range
betweenness	30.40	156.53	391.70	0.00–5323.65
closeness	0.23	0.24	0.11	0.02–1.00
clustering coefficient	0.13	0.14	0.07	0.00–1.00
degree	2.00	4.61	7.54	1.00–153.00
eigenvalue centrality	0.00	0.04	0.16	0.00–1.00

**Figure 4.**
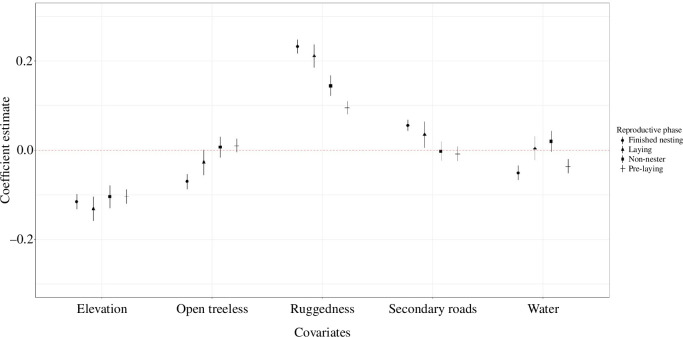
Coefficient plot depicting roost site selection during reproductive phases of female eastern wild turkey (*M. gallopavo silvestris*) across the southeastern United States during 2014–2021. The whiskers depict 95% confidence intervals around mean estimates.

The global model (*w_i_* = 1.00; table 6) best fitted our data for predicting roost site selection. Females selected for roost sites were at lower elevations and with greater ruggedness (figure 4). During the pre-laying, females selected roost sites closer to water, whereas during laying they selected roosts further from secondary roads (figure 4). When females ceased reproductive activities, they selected roost sites closer to open treeless areas and water but further from secondary roads (figure 4).

**Table 6.** Akaike’s iinformation criterion with small sample bias adjustment (AICc), number of parameters (*K*), ΔAICc, adjusted Akaike weight of evidence (*w*_*i*_) in support of model and log-likelihood (LL) for each model examining roost site selection and selection of hub versus satellite roosts of female eastern wild turkeys (*M. gallopavo silvestris*) across the southeastern United States during 2014–2021.

model	*K*	AICc	ΔAICc	*w*_*i*_	LL
roost site selection					
global	22	353 563.9	0.00	1.00	−176 760.0
resource	14	354 977.0	1413.1	0.00	−177 474.5
feature	10	357 507.3	3943.4	0.00	−178 743.7
(.)	2	358 816.5	5252.6	0.00	−179 406.3
hub versus satellite roosts					
global	22	52 304.3	0.00	1.00	−26 130.1
resource	14	53 324.4	20.4	0.00	−26 148.4
feature	10	52 944.0	639.7	0.00	−26 462.0
(.)	2	52 960.4	656.1	0.00	−26 478.2

**Figure 5.**
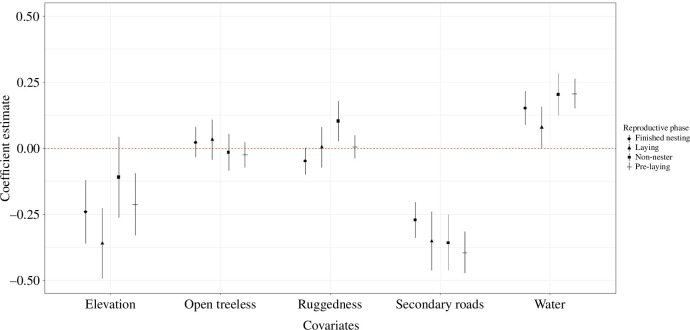
Coefficient plot depicting hub versus satellite site during the reproductive phase of female eastern wild turkey (*M. gallopavo silvestris*) across the southeastern United States during 2014–2021. The whiskers depict 95% confidence intervals around mean estimates.

The global model (*w*_*i*_ = 1.00; table 6) best fitted our data for predicting selection of hub versus satellite roosts. During all phases, females selected hub roosts that were closer to secondary roads and further from water (figure 5). Additionally, during pre-laying, laying and when the reproductive season ceased, females selected for hub roosts that were at lower elevations. Non-nesting females selected for hub roosts with greater ruggedness (figure 5).

## 4. Discussion

We observed high site fidelity and lower distances between consecutive roost sites for female wild turkeys during the reproductive season, observations that differ markedly from roost behaviours exhibited by males (table 4; [32,43]). This has also been observed in capercaillie (*Tetrao urogallus*), an upland bird that relies on similar reproductive and resource acquisition strategies [88]. With capercaillie and probably with female turkeys, remaining at the same roost sites during the breeding season could be a tactic used to increase exposure and predictability to males while also increasing resource acquisition, which is crucial for egg laying and incubation [89].

We found that the roost site network evaluated for individual female wild turkeys had few highly centralized hub roosts (approx. 13%) that had many connections, promoting connectivity to other roosts in the network. Maintaining networks with centralized hub sites allows an individual to learn about the location and exploit high-quality resources [90,91]. Hence, networks exhibiting these characteristics are often associated with resource-rich areas [21,22] , interactions with conspecifics for information [92–94] or mate acquisition [88,95]. The level of experience an individual has with the landscape has been attributed to reduced predation risk by identifying refuges and escape routes [96,97]. For example, pheasants (*Phasianus colchicus*) killed by predators were more on the periphery of their home range than towards the centre [98]. Hence, female wild turkeys maintaining hub roosts centrally located within their reproductive range may facilitate increased vigilance and efficient resource acquisition that positively influences fitness.

Environmental variation across landscapes has the potential to influence how species interact with terrain [99,100]. For wild turkeys that occur within forested landscapes, potential roost sites are often ubiquitous within individual home ranges [32]. By contrast, for subspecies residing in more arid environments (such as Gould’s (*M. gallopavo Mexicana*), Merriam’s (*M. gallopavo merriami*) and Rio Grande wild turkeys (*M. gallopavo intermedia*)), roost sites are often confined to riparian corridors [101–104]. We found that roosts selected by female wild turkeys were at lower elevations with greater ruggedness regardless of reproductive phase. This is consistent not only with eastern wild turkeys but Gould’s, Merriam’s and Rio Grande wild turkeys that select roosts at lower elevations often associated with riparian corridors [43,101,102,104]. Increased ruggedness is often associated with the irregularity on the outer edges of riparian corridors where the landscape begins to become steep [99]. Selecting roosts in areas with greater ruggedness enables avian species to perch at elevated positions, enhancing their ability to better observe their surroundings and increase the efficiency of flights [105,106]. Wild turkeys depend on their vision to detect predators before flying down from their roosts in the morning [57], thus, choosing roost sites in areas with increased ruggedness may diminish the risk of predation.

Resource acquisition is necessary for the survival of avian species upon completion of the reproductive period [107]. We found that upon the completion of reproduction, female wild turkeys selected to roost closer to open treeless areas and water. Open treeless areas are selected by wild turkeys throughout portions of their annual cycle [46,47,49], and provide the opportunity to increase foraging efficiency while also increasing the detectability of predators, which has similarly been shown in various Galliformes [108,109]. Furthermore, open treeless areas provide forb and grass communities that provide relatively high densities of invertebrates necessary for poult development [49,110–112]. Additionally, selecting roost sites closer to water could buffer thermal extremes common throughout the southeastern United States during the summer after reproductive activities cease [68,113,114].

Locations of hub nodes within a network are often affiliated with greater connectivity to other nodes within the network [19]. Within our network, hub roosts were situated closer to secondary roads, which may provide quality foraging opportunities and opportunities to escape predation threats [68,113] and enhance mobility for females [110]. Furthermore, the low-intensity maintenance of trails and secondary roads promotes early successional vegetative communities, which were spatially limited on our study sites [68]. Research on hazel grouse (*Tetrastes bonasia*) has shown that in areas with minimal trail maintenance, individuals may select trails to exploit early successional plant species [115].

Few ecological studies have explored the impact of environmental characteristics and resources on spatial networks of wildlife [22]. Our findings indicate that wild turkeys develop and maintain a spatial network of roosts within their reproductive range, with centralized hub roosts that are ecologically important. The roost site networks used by wild turkeys require further study into how conspecific interactions may occur, as extant literature demonstrates the critical role of networks in breeding [116], resource access [21,117] and disease or parasite transmission [118–120] among various gregarious species.

## Data Availability

Data and relevant code and data for this research work are stored in Dyrad [121].

## References

[1] Hartse KM . 1994 Sleep in insects and Nonmammalian vertebrates. In Principles and practice of sleep medicine (eds MH Kryger , T Roth , WC Dement ), pp. 95–104, 2nd edn. Philadelphia, PA: Elsevier.

[2] Tobler I . 2011 Phylogeny of sleep regulation. In Principles and practice of sleep medicine (eds MH Kryger , T Roth , WC Dement ), pp. 77–90. Philadelphia, PA: Elsevier. (10.1016/B0-72-160797-7/50014-8)

[3] Lima SL , Rattenborg NC , Lesku JA , Amlaner CJ . 2005 Sleeping under the risk of predation. Anim. Behav. **70** , 723–736. (10.1016/j.anbehav.2005.01.008)

[4] Rattenborg NC , Amlaner CJ . 2002 Phylogeny of sleep. In Sleep medicine (eds TL Lee-Chiong , MJ Sateia , MA Carskadon ), pp. 7–22, 1st edn. Philadelphia, PA: Hanley and Belfus.

[5] Zepelin H , Rechtschaffen A . 1974 Mammalian sleep, longevity, and energy metabolism. Brain Behav. Evol. **10** , 425–470. (10.1159/000124330) 4464027

[6] Amlaner CJ , Ball NJ . 1983 A synthesis of sleep in wild birds. Behaviour **87** , 85–119. (10.1163/156853983X00138)

[7] Singhal S , Johnson MA , Ladner JT . 2007 The behavioral ecology of sleep: natural sleeping site choice in three Anolis lizard species. Behaviour **144** , 1033–1052. (10.1163/156853907781871860)

[8] Stiles FG , Skutch AF . 1989 Guide to the birds of Costa Rica. Ithaca, NY: Cornell University Press.

[9] Webber QMR , Albery GF , Farine DR , Pinter-Wollman N , Sharma N , Spiegel O , Vander Wal E , Manlove K . 2023 Behavioural ecology at the spatial-social interface. Biol. Rev. **98** , 868–886. (10.1111/brv.12934) 36691262

[10] Bakken GS . 1992 Measurement and application of operative and standard operative temperatures in ecology. Am. Zool. **32** , 194–216. (10.1093/icb/32.2.194)

[11] Franklin SP . 2004 Predator influence on golden lion Tamarin nest choice and presleep behavior. MSc thesis, University of Maryland, College Park, MD, USA.

[12] Smith AC , Knogge C , Huck M , Löttker P , Buchanan-Smith HM , Heymann EW . 2007 Long-term patterns of sleeping site use in wild saddleback (Saguinus fuscicollis) and mustached tamarins (S. mystax): effects of foraging, thermoregulation, predation, and resource defense constraints. Am. J. Phys. Anthropol. **134** , 340–353. (10.1002/ajpa.20676) 17632801

[13] Di Bitetti MS , Vidal EML , Baldovino MC , Benesovsky V . 2000 Sleeping site preferences in tufted capuchin monkeys (Cebus apella nigritus). Am. J. Primatol. **50** , 257–274. (10.1002/(SICI)1098-2345(200004)50:4<257::AID-AJP3>3.0.CO;2-J) 10768350

[14] Li D , Zhou Q , Tang X , Huang H , Huang C . 2011 Sleeping site use of the white-headed langur Trachypithecus leucocephalus: the role of predation risk, territorial defense, and proximity to feeding sites. Curr. Zool. **57** , 260–268. (10.1093/czoolo/57.3.260)

[15] Giraldeau LA , Dubois F . 2008 Social foraging and the study of exploitative behavior. Adv. Study Behav. **38** , 59–104. (10.1016/S0065-3454(08)00002-8)

[16] Maldonado‐Chaparro AA , Montiglio P , Forstmeier W , Kempenaers B , Farine DR . 2018 Linking the fine‐scale social environment to mating decisions: a future direction for the study of extra‐pair paternity. Biol. Rev. **93** , 1558–1577. (10.1111/brv.12408) 29533010

[17] Creel S , Schuette P , Christianson D . 2014 Effects of predation risk on group size, vigilance, and foraging behavior in an African ungulate community. Behav. Ecol. **25** , 773–784. (10.1093/beheco/aru050)

[18] Newman ME . 2003 The structure and function of complex networks. SIAM Rev. **45** , 167–256. (10.1137/S003614450342480)

[19] Krause J , James R , Franks DW , Croft DP . 2015 Animal social networks. Oxford, UK: Oxford University Press. (10.1093/acprof:oso/9780199679041.001.0001)

[20] Prima M , Duchesne T , Fortin A , Rivest L , Drapeau P , St‐Laurent M , Fortin D . 2019 A landscape experiment of spatial network robustness and space‐use reorganization following habitat fragmentation. Funct. Ecol. **33** , 1663–1673. (10.1111/1365-2435.13380)

[21] Watts BD , Dyer RJ . 2018 Structure and resilience of bald eagle roost networks. Wildl. Soc. Bull. **42** , 195–203. (10.1002/wsb.865)

[22] le Roux CE , Nocera JJ . 2021 Roost sites of chimney swift (Chaetura pelagica) form large-scale spatial networks. Ecol. Evol. **11** , 3820–3829. (10.1002/ece3.7235) 33976777 PMC8093691

[23] Woods CP , Czenze ZJ , Brigham RM . 2019 The avian “hibernation” enigma: thermoregulatory patterns and roost choice of the common poorwill. Oecologia **189** , 47–53. (10.1007/s00442-018-4306-0) 30460539

[24] Yuan B , Yan Y , Cheng Z , Jiang A . 2018 Roosting habitat selection of Hume’s pheasant (Syrmaticus humiae) in a fragmented forest patch, northwestern Guangxi, southwestern China. Glob. Ecol. Conserv. **16** , e00457. (10.1016/j.gecco.2018.e00457)

[25] Cody ML . 1985 Habitat selection in birds. New York, NY: Academic Press.

[26] Thiel D , Unger C , Kéry M , Jenni L . 2007 Selection of night roosts in winter by Capercaillie tetrao urogallus in Central Europe. Wildl. Biol. **13** , 73–86. (10.2981/0909-6396(2007)13[73:SONRIW]2.0.CO;2)

[27] Beauchamp G . 1999 The evolution of communal roosting in birds: origin and secondary losses. Behav. Ecol. **10** , 675–687. (10.1093/beheco/10.6.675)

[28] De-Jun K , Xiao-Jun Y , Qiang L , Xing-Yao Z , Jun-Xing Y . 2011 Winter habitat selection by the vulnerable black-necked crane Grus nigricollis in Yunnan, China: implications for determining effective conservation actions. Oryx **45** , 258–264. (10.1017/S0030605310000888)

[29] Zoghby BA , Little RM , Ryan PG , Hockey PA . 2016 Patterns of roost site selection and use by southern ground-hornbills in north-eastern South Africa. Ostrich **87** , 125–130. (10.2989/00306525.2016.1156180)

[30] Rosenberg DK , McKelvey KS . 1999 Estimation of habitat selection for central-place foraging animals. J. Wildl. Manage. **63** , 1028. (10.2307/3802818)

[31] Chaverri G . 2010 Comparative social network analysis in a leaf-roosting bat. Behav. Ecol. Sociobiol. **64** , 1619–1630. (10.1007/s00265-010-0975-3)

[32] Byrne M , Collier B , Chamberlain M . 2015 Roosting behavior of male eastern and Rio Grande wild turkeys. Natl Wild Turkey Symp. **11** , 175–185.

[33] Austin PD , DeGraff LW . 1975 Winter survival of wild turkeys in the southern Adirondacks. Natl Wild Turkey Symp. **3** , 55–60.

[34] Vander Haegen WM , Sayre MW , Dodge WE . 1989 Winter use of agricultural habitats by wild turkeys in Massachusetts. J. Wildl. Manage. **53** , 30–33. (10.2307/3801300)

[35] Flake LD , Craft RA , Tucker WL . 1995 Vegetation characteristics of wild turkey roost sites during summer in south-central South Dakota. Natl Wild Turkey Symp. **7** , 159–164.

[36] Mackey DL . 1984 Roosting habitat of Merriam’s turkeys in south-central Washington. J. Wildl. Manage. **48** , 1377–1382. (10.2307/3801801)

[37] Swearingin RM , Butler MJ , Ballard WB , Wallace MC , Phillips RS , Walker RN , McKenzie-Damron S , Ruthven DC . 2011 Winter roost characteristics of Rio Grande wild turkeys in the rolling plains of Texas. Natl Wild Turkey Symp. **10** , 265–277.

[38] Chamberlain MJ , Leopold BD , Burger LW . 2000 Characteristics of roost sites of adult wild turkey females. J. Wildl. Manage **64** , 1025–1032. (10.2307/3803213)

[39] Frary VJ , Ingraldi MF , Sesnie SE , Horncastle, V , Reichold Z . 2011 Landscape-scale identification of Merriam’s wild turkey roosting habitat in a managed ponderosa pine forest. Natl Wild Turkey Symp. **10** , 293–300.

[40] Keegan TW , Crawford JA . 2005 Roost habitat selection by Rio Grande turkeys in Oregon. Natl Wild Turkey Symp. **9** , 253–259.

[41] Perlichek KB , Harveson LA , Warnock BJ , Tarrant B . 2009 Habitat characteristics of winter roost sites of wild turkeys in Trans-Pecos, Texas. Southwest Nat. **54** , 446–452. (10.1894/MH-42.1)

[42] Phillips CE , Demaso SJ , Kuvlesky WP , Hewitt DG . 2011 Landscape metrics related to Rio Grande wild turkey winter roosts in south Texas. Natl Wild Turkey Symp. **10** , 251–264.

[43] Wakefield CT , Martin JA , Wightman PH , Bond BT , Lowrey DK , Cohen BS , Collier BA , Chamberlain MJ . 2020 Hunting activity effects on roost selection by male wild turkeys. J. Wildl. Manag. **84** , 458–467. (10.1002/jwmg.21812)

[44] Miller DA , Conner LM . 2007 Habitat selection of female turkeys in a managed pine landscape in Mississippi. J. Wildl. Manag. **71** , 744–751. (10.2193/2005-738)

[45] Chamberlain M , Leopold B . 1998 Microhabitat characteristics of wild turkey prenest and nest site selection in central Mississippi. In Proc. of the Southeastern Association of Fish and Wildlife Agencies, vol. 52, pp. 274–282,

[46] Bakner NW , Cohen BS , Collier BA , Chamberlain MJ . 2022 Recursive movements of eastern wild turkey broods in the southeastern United States. Wildl. Soc. Bull. **46** , e1274. (10.1002/wsb.1274)

[47] Bakner NW , Fyffe N , Oleson B , Smallwood A , Heffelfinger JR , Chamberlain MJ , Collier BA . 2022 Roosting ecology of Gould’s wild turkeys in southeastern Arizona. J. Wildl. Manag. **86** , e22277. (10.1002/jwmg.22277) PMC10616159 37903898

[48] Bakner NW , Schofield LR , Cedotal C , Chamberlain MJ , Collier BA . 2019 Incubation recess behaviors influence nest survival of wild turkeys. Ecol. Evol. **9** , 14053–14065. (10.1002/ece3.5843) 31938503 PMC6953688

[49] Chamberlain MJ , Cohen BS , Bakner NW , Collier BA . 2020 Behavior and movement of wild turkey broods. J. Wildl. Manage. **84** , 1139–1152. (10.1002/jwmg.21883)

[50] Lohr AK , Martin JA , Wann GT , Cohen BS , Collier BA , Chamberlain MJ . 2020 Behavioral strategies during incubation influence nest and female survival of wild turkeys. Ecol. Evol. **10** , 11752–11765. (10.1002/ece3.6812) 33144998 PMC7593161

[51] Ulrey EE , Chamberlain MJ , Collier BA . 2023 Reproductive asynchrony within social groups of female eastern wild turkeys. Ecol. Evol. **13** , e10171. (10.1002/ece3.10171) 37325717 PMC10266966

[52] Schrocder MA , White GC . 1993 Dispersion of greater prairie chicken nests in relation to lek location: evaluation of the hot-spot hypothesis of lek evolution. Behav. Ecol. **4** , 266–270. (10.1093/beheco/4.3.266)

[53] Westcott DA . 1997 Lek locations and patterns of female movement and distribution in a neotropical frugivorous bird. Anim. Behav. **53** , 235–247. (10.1006/anbe.1996.0294)

[54] Conley MD , Yeldell NA , Chamberlain MJ , Collier BA . 2016 Do movement behaviors identify reproductive habitat sampling for wild turkeys? Ecol. Evol. **6** , 7103–7112. (10.1002/ece3.2401) 28725385 PMC5513226

[55] Bakner NW , Ulrey EE , Collier BA , Chamberlain MJ . 2024 Prospecting during egg laying informs incubation recess movements of eastern wild turkeys. Mov. Ecol. **12** , 4. (10.1186/s40462-024-00451-3) 38229127 PMC10792941

[56] Schofield LR . 2019 Evaluation of reproductive phenology and ecology of wild turkey (Meleagris Gallopavo) across the southeastern United States. Thesis, Louisiana State University, Baton Rouge, LA, USA.

[57] Pelham PH , Dickson JG . 1992 Physical characteristics. In The wild turkey: biology and management, vol. 32 (ed. JG Dickson ), pp. 32–45. Mechanicsburg, PA: Stackpole Books.

[58] Guthrie JD et al . 2011 Evaluation of a global positioning system backpack transmitter for wild turkey research. J. Wildl. Manag. **75** , 539–547. (10.1002/jwmg.137)

[59] Cohen BS , Prebyl TJ , Collier BA , Chamberlain MJ . 2018 Home range estimator method and GPS sampling schedule affect habitat selection inferences for wild turkeys. Wildl. Soc. Bull. **42** , 150–159. (10.1002/wsb.845)

[60] R Development Core Team . 2023 *R: a language and environment for statistical computing* . Vienna, Austria: R Foundation for Statistical Computing.

[61] Gupte PR , Beardsworth CE , Spiegel O , Lourie E , Toledo S , Nathan R , Bijleveld AI . 2022 A guide to pre-processing high-throughput animal tracking data. J. Anim. Ecol. **91** , 287–307. (10.1111/1365-2656.13610) 34657296 PMC9299236

[62] Conley M , Oetgen J , Barrow J , Chamberlain M , Skow K , Collier B . 2015 Habitat selection, incubation, and incubation recess ranges of nesting female Rio Grande wild turkeys in Texas. Natl Wild Turkey Symp. **11** , 117–126.

[63] Collier BA , Fyffe N , Smallwood A , Oleson B , Bakner NW , Heffelfinger JR , Chamberlain MJ . 2019 Reproductive ecology of Gould’s wild turkeys (Meleagris gallopavo mexicana) in Arizona. Wilson. J. Ornithol. **131** , 667–679. (10.1676/18-162)

[64] Moscicki DJ , White JH , Hardin JB , Chamberlain MJ , Collier BA . 2023 Phenology‐specific space use by Rio Grande wild turkeys. J. Wildl. Manag. **87** , e22331. (10.1002/jwmg.22331)

[65] Hahsler M , Piekenbrock M , Doran D . 2019 dbscan: fast density-based clustering with R. J. Stat. Softw. **91** , 1–30. (10.18637/jss.v091.i01)

[66] Cohen BS , Oleson B , Fyffe N , Smallwood A , Bakner N , Nelson S , Chamberlain MJ , Collier BA . 2022 Movement, spatial ecology, and habitat selection of translocated Gould’s wild turkeys. Wildl. Soc. Bull. **46** , e1270. (10.1002/wsb.1270)

[67] Gerrits AP , Wightman PH , Cantrell JR , Ruth C , Chamberlain MJ , Collier BA . 2020 Movement ecology of spring wild turkey hunters on public lands in South Carolina, USA. Wildl. Soc. Bull. **44** , 260–270. (10.1002/wsb.1094)

[68] Nelson SD , Keever AC , Wightman PH , Bakner NW , Collier BA , Chamberlain MJ , Cohen BS . 2023 Age‐based shifts in habitat selection of wild turkey broods. J. Wildl. Manag. **87** , e22494. (10.1002/jwmg.22494)

[69] Croft DP , James R , Krause J . 2008 Exploring animal social networks. Princeton, NJ: Princeton University Press. (10.1515/9781400837762)

[70] Farine DR . 2015 Proximity as a proxy for interactions: issues of scale in social network analysis. Anim Behav. **104** , e1–e5. (10.1016/j.anbehav.2014.11.019)

[71] Farine DR , Whitehead H . 2015 Constructing, conducting and interpreting animal social network analysis. J. Anim. Ecol. **84** , 1144–1163. (10.1111/1365-2656.12418) 26172345 PMC4973823

[72] Proulx SR , Promislow DE , Phillips PC . 2005 Network thinking in ecology and evolution. Trends Ecol. Evol. **20** , 345–353. (10.1016/j.tree.2005.04.004) 16701391

[73] Csardi G , Nepusz T . 2006 The igraph software package for complex network research. Int. J. Complex Syst **1695** , 1–9. http://igraph.org

[74] Freeman LC , Borgatti SP , White DR . 1991 Centrality in valued graphs: a measure of betweenness based on network flow. Soc. Networks **13** , 141–154. (10.1016/0378-8733(91)90017-N)

[75] Bavelas A . 1950 Communication patterns in task oriented groups. J. Acoust. Soc. Am **22** , 725–730. (10.1121/1.1906679)

[76] Borgatti SP , Everett MG . 2006 A graph-theoretic perspective on centrality. Soc. Networks **28** , 466–484. (10.1016/j.socnet.2005.11.005)

[77] Dormann CF et al . 2013 Collinearity: a review of methods to deal with it and a simulation study evaluating their performance. Ecography **36** , 27–46. (10.1111/j.1600-0587.2012.07348.x)

[78] Magnússon A , Skaug HJ , Nielsen A , Berg CW , Kristensen K , Maechle M , Van Bentham K , Bolker B , Brooks ME . 2017 *glmmTMB: generalized linear mixed models using template model builder* *.* R package version 0.1.3. See https://github.com/glmmTMB.

[79] Kranstauber B , Kays R , Lapoint SD , Wikelski M , Safi K . 2012 A dynamic Brownian bridge movement model to estimate utilization distributions for heterogeneous animal movement. J. Anim. Ecol. **81** , 738–746. (10.1111/j.1365-2656.2012.01955.x) 22348740

[80] Byrne ME , Guthrie JD , Hardin J , Collier BA , Chamberlain MJ . 2014 Evaluating wild turkey movement ecology: an example using first‐passage time analysis. Wildl. Soc. Bull. **38** , 407–413. (10.1002/wsb.404)

[81] Manly BFJ , McDonald LL , Thomas DL , McDonald TL , Erickson WP . 2002 Resource selection by animals: statistical design and analysis for field studies, 2nd edn. Dordrecht, The Netherlands: Kluwer Academic.

[82] Benson JF . 2013 Improving rigour and efficiency of use‐availability habitat selection analyses with systematic estimation of availability. Methods Ecol. Evol. **4** , 244–251. (10.1111/2041-210x.12006)

[83] Johnson CJ , Nielsen SE , Merrill EH , Mcdonald TL , Boyce MS . 2006 Resource selection functions based on use–availability data: theoretical motivation and evaluation methods. J. Wildl. Manage. **70** , 347–357. (10.2193/0022-541X(2006)70[347:RSFBOU]2.0.CO;2)

[84] Muff S , Signer J , Fieberg J . 2020 Accounting for individual-specific variation in habitat-selection studies: efficient estimation of mixed-effects models using Bayesian or frequentist computation. J. Anim. Ecol. **89** , 80–92. (10.1111/1365-2656.13087) 31454066

[85] Gelman A . 2008 Scaling regression inputs by dividing by two standard deviations. Stat. Med. **27** , 2865–2873. (10.1002/sim.3107) 17960576

[86] Akaike H . 1973 Information theory and an extension of the maximum likelihood principle. In Proc. Int. Symp. Inform. Theory, vol. 2, pp. 267–281, Kiadó, Budapest: Akad.

[87] Anderson DR , Burnham KP . 2002 Avoiding pitfalls when using informatheoretic methtion-theoretic methods. J. Wildl. Manage. **66** , 912–918. (10.2307/3803155)

[88] Gjerde I , Wegge P , Rolstad J . 2000 Lost hotspots and passive female preference: the dynamic process of lek formation in capercaillie Tetrao urogallus . Wildl. Biol. **6** , 291–298. (10.2981/wlb.2000.029)

[89] Gerber BD , Hooten MB , Peck CP , Rice MB , Gammonley JH , Apa AD , Davis AJ . 2019 Extreme site fidelity as an optimal strategy in an unpredictable and homogeneous environment. Funct. Ecol. **33** , 1695–1707. (10.1111/1365-2435.13390)

[90] Bracis C , Gurarie E , Van Moorter B , Goodwin RA . 2015 Memory effects on movement behavior in animal foraging. PLoS ONE **10** , e0136057. (10.1371/journal.pone.0136057) 26288228 PMC4542208

[91] Spencer WD . 2012 Home ranges and the value of spatial information. J. Mammal. **93** , 929–947. (10.1644/12-MAMM-S-061.1)

[92] Dermody BJ , Tanner CJ , Jackson AL . 2011 The evolutionary pathway to obligate scavenging in Gyps vultures. PLoS ONE **6** , e24635. (10.1371/journal.pone.0024635) 21931786 PMC3169611

[93] Ruxton GD . 1995 Foraging in flocks: non-spatial models may neglect important costs. Ecol. Modell. **82** , 277–285. (10.1016/0304-3800(94)00098-3)

[94] Ruxton GD , Houston DC . 2002 Modelling the energy budget of a colonial bird of prey, the Ruppell’s griffon vulture, and consequences for its breeding ecology. Afr. J. Ecol. **40** , 260–266. (10.1046/j.1365-2028.2002.00368.x)

[95] Alonso JC , Morales MB , Alonso JA . 2000 Partial migration, and Lek and nesting area fidelity in female great bustards. Condor **102** , 127–136. (10.1093/condor/102.1.127)

[96] Gaynor KM , Brown JS , Middleton AD , Power ME , Brashares JS . 2019 Landscapes of fear: spatial patterns of risk perception and response. Trends Ecol. Evol. **34** , 355–368. (10.1016/j.tree.2019.01.004) 30745252

[97] Laundré JW , Hernández L , Ripple WJ . 2010 The landscape of fear: ecological implications of being afraid. Open Ecol. J. **3** , 1–7. (10.2174/1874213001003030001)

[98] Heathcote RJP , Whiteside MA , Beardsworth CE , Van Horik JO , Laker PR , Toledo S , Orchan Y , Nathan R , Madden JR . 2023 Spatial memory predicts home range size and predation risk in pheasants. Nat. Ecol. Evol. **7** , 461–471. (10.1038/s41559-022-01950-5) 36690732

[99] Chapin FS , Matson PA , Mooney HA , Vitousek PM . 2002 Principles of terrestrial ecosystem ecology. New York, NY: Springer-Verlag. (10.1007/b97397)

[100] Whittaker RH . 1967 Gradient analysis of vegetation. Biol. Rev. **42** , 207–264. (10.1111/j.1469-185x.1967.tb01419.x) 4859903

[101] Beasom SL , Wilson D . 1992 Rio Grande turkey. In The wild turkey: biology and management (ed. JG Dickson ), pp. 129–143. Harrisburg, PA: Stackpole Books.

[102] Crockett BC . 1973 Quantitative evaluation of winter roost sites of the Rio Grande Turkey in North-central Oklahoma. In Wild Turkey management: current problems and programs (eds GC Sanderson , HC Schultz ), pp. 211–218. Columbia, MO: University of Missouri Press.

[103] Márquez Olivas M , García Moya E , González-rebeles Islas C , Vaquera Huerta H . 2007 Roost sites characteristics of wild turkey (Meleagris gallopavo mexicana) in Sierra Fria, Aguascalientes, Mexico. Rev. Mex. Biodiv. **78** , 163–173. (10.22201/ib.20078706e.2007.001.391)

[104] Schemnitz SD , Zornes ML . 1995 Management practices to benefit Gould’s Turkey in the Peloncillo mountains, New Mexico. In Biodiversity and management of the Madrean archipelago: the sky islands of Southwestern United States and Northwestern Mexico. United States Department of Agriculture Forest service general technical report RM-GTR-264 (eds LF DeBano , PF Folliott , A Ortega-Rubio , GJ Gottfried , RH Hamre , CB Edminster ). Fort Collins, CO: United States Department of Agriculture Forest Service.

[105] Domenech R , Bedrosian BE , Crandall RH , Slabe VA . 2015 Space use and habitat selection by adult migrant golden eagles wintering in the Western United States. J. Raptor Res. **49** , 429–440. (10.3356/rapt-49-04-429-440.1)

[106] Watson JW , Duff AA , Davies RW . 2014 Home range and resource selection by GPS-monitored adult golden eagles in the Columbia Plateau ecoregion: Implications for wind power development. J. Wildl. Manage. **78** , 1012–1021. (10.1002/jwmg.745)

[107] Deeming DC , Reynolds SJ . 2015 Nests, eggs, and incubation: new ideas about avian reproduction. Oxford, UK: Oxford University Press.

[108] Aldridge CL , Boyce MS . 2007 Linking occurrence and fitness to persistence: habitat-based approach for endangered greater sage-grouse. Ecol. Appl. **17** , 508–526. (10.1890/05-1871) 17489256

[109] Storch I . 1994 Habitat and survival of Capercaillie tetrao urogallus nests and broods in the Bavarian alps. Biol. Conserv. **70** , 237–243. (10.1016/0006-3207(94)90168-6)

[110] Bakner NW , Collier BA , Chamberlain MJ . 2023 Behavioral-dependent recursive movements and implications for resource selection. Sci. Rep. **13** , 16632. (10.1038/s41598-023-43907-z) 37789205 PMC10547709

[111] Healy WM . 1985 Turkey poult feeding activity, invertebrate abundance, and vegetation structure. J. Wildl. Manage. **49** , 466. (10.2307/3801553)

[112] Rumble MA , Anderson SH . 1996 Feeding ecology of Merriam’s turkeys (Meleagris gallopavo merriami) in the Black Hills, South Dakota. Am. Midl. Nat. **136** , 157–171. (10.2307/2426641)

[113] Nelson SD , Keever AC , Wightman PH , Bakner NW , Argabright CM , Byrne ME , Collier BA , Chamberlain MJ , Cohen BS . 2022 Fine‐scale resource selection and behavioral tradeoffs of eastern wild turkey broods. J. Wildl. Manage. **86** , e22222. (10.1002/jwmg.22222)

[114] Smith EK , O’Neill J , Gerson AR , Wolf BO . 2015 Avian thermoregulation in the heat: resting metabolism, evaporative cooling and heat tolerance in Sonoran Desert doves and quail. J. Exp. Biol. **218** , 3636–3646. (10.1242/jeb.128645) 26582934

[115] Matysek M , Gwiazda R , Bonczar Z . 2020 The importance of habitat diversity and plant species richness for hazel grouse occurrence in the mixed mountain forests of the Western Carpathians. Eur. J. Forest Res. **139** , 1057–1065. (10.1007/s10342-020-01307-2)

[116] Ryder TB , Blake JG , Parker PG , Loiselle BA . 2011 The composition, stability, and kinship of reproductive coalitions in a lekking bird. Behav. Ecol. **22** , 282–290. (10.1093/beheco/arq213)

[117] Peignier M , Webber QM , Koen EL , Laforge MP , Robitaille AL , Vander Wal E . 2019 Space use and social association in a gregarious ungulate: testing the conspecific attraction and resource dispersion hypotheses. Ecol. Evol. **9** , 5133–5145. (10.1002/ece3.5071) 31110667 PMC6509382

[118] Fortuna MA , Popa-Lisseanu AG , Ibáñez C , Bascompte J . 2009 The roosting spatial network of a bird-predator bat. Ecology **90** , 934–944. (10.1890/08-0174.1) 19449689

[119] Guimarães Jr PR , de Menezes MA , Baird RW , Lusseau D , Guimarães P , dos Reis SF . 2007 Vulnerability of a killer whale social network to disease outbreaks. Phys. Rev. E **76** , 042901. (10.1103/PhysRevE.76.042901) 17995045

[120] White LA , Forester JD , Craft ME . 2017 Using contact networks to explore mechanisms of parasite transmission in wildlife. Biol. Rev. **92** , 389–409. (10.1111/brv.12236) 26613547

[121] Bakner NW , Ulrey EE , Wightman PH , Gulotta NA , Collier BA , Chamberlain MJ . 2024 Data for: Spatial roost networks and resource selection of female wild turkeys. Dryad (10.5061/dryad.gxd2547sw) PMC11285678 39076792

